# Flavor Compounds Identification and Reporting

**DOI:** 10.3390/molecules31020249

**Published:** 2026-01-12

**Authors:** Henryk H. Jeleń

**Affiliations:** Faculty of Food Science and Nutrition, Poznań University of Life Sciences, Wojska Polskiego 31, 60-624 Poznań, Poland; henryk.jelen@up.poznan.pl

## 1. Isolation of Volatile Compounds from the Matrix

Both exhaustive and non-exhaustive extraction methods can be used in the isolation of volatile compounds. Researchers should be aware of the fundamentals, advances, and drawbacks of the extraction methods they use. For the isolation of essential oils, authors should refer to [1].

## 2. Volatile Compounds Identification

Gas chromatography–mass spectrometry (GC–MS) is usually the technique of choice for the identification of volatile compounds. For known compounds, their identification should be based on full mass spectra and retention indices. Preferably, the analyses should be run on two columns of an orthogonal separation mechanism (usually DB-5 and Wax-type stationary phases).

For unequivocal identification of a volatile compound, its mass spectra and retention indices should be compared with an authentic standard analyzed in the same conditions. In written work, authors should note which compounds were identified based on these criteria. When no authentic standard confirms the identity of a compound, it should be classified as tentatively identified.

For optical isomers/compounds, no optical rotation/absolute configuration should be provided if no chiral chromatography is used for enantiomer separation. If commercially available chiral compound is used, its source should be specified. It is recommended that chromatograms that show the separated enantiomers of individual compounds are presented in the Supplementary Materials.

Unequivocal identification of Z,E-isomers can be problematic, as their spectra are often indistinguishable and their retention data are also often similar.

When a compound is described for the first time, the ^1^H, ^13^C, HSQC, HMBC NMR, IR, and HRMS spectra should be provided to elucidate its structure.

Retention indices based on alkanes for a particular column phase are crucial in the identification of volatile compounds. Retention indices provided by authors should be compared to data from databases (such as NIST) and/or the literature, i.e., indices of authentic substances measured by other authors (e.g., [2]). In comprehensive two-dimensional chromatography (GC×GC), retention indices calculated for a first column can be used for identification [3].

To determine odor-active compounds within a mixture of volatile compounds, the use of gas chromatography–olfactometry (GC–O) is recommended [4].

## 3. Quantitation of Volatile Compounds

For the expression of the composition of volatile compounds mixture in an area% of components (with the simplified assumption that the detector responds equally to all components), TIC (total ion current) peak areas should be used when analyses are performed using GC–MS. Area % can be provided using an FID detector when compounds have been identified by GC–MS using the same column in the same analytical conditions. Authors should be aware that the area% expression of a volatile compound mixture depends significantly on the isolation method used. This is especially important for sorbent-based extraction methods.

For quantitative analysis of aroma compounds, SIDA (stable isotope dilution analysis) is preferred, where an isotopologue (^2^H, ^13^C, ^15^N) of a particular compound is added before sample preparation process. The similarity of the isotopically labelled analogue with the determined compound provides comparable losses during the sample preparation process. In the preparation and storage of deuterated standards, D/H exchange should be monitored. For SIDA, a mass spectrometer is used as a detector, and the number of labelled atoms should preferably be more than one.

If the isotopologues of the analyzed standards are unavailable, then the standard addition method is preferred taking into account the complexity of a food as a matrix. Only in simple matrices can external calibration be performed. Authors should be aware of the influence of matrix constituents on calibration. Special care should be taken for complex non-heterogenic matrices, solid foods, etc.

When conducting quantitative analyses especially with SPME, one must account for matrix composition [5,6,7]. Quantitative SPME analysis should always be based on the calibration curves for a particular compound [8,9]. The determination of relative quantities by SPME does not permit a comparison of the quantities of different compounds in the same samples. Constant sampling conditions should be used in SPME sampling for comparative purposes (relative quantities).

## 4. Non-Volatile Compound Identification

Liquid chromatography (LC) is utilized in non-volatile compound separation, whereas LC–MS is used for the identification of unknown compounds. Considering the character of LC–MS mass spectra, appropriate precautions should be taken [10].

Profiling and quantification of non-volatile compounds could also be performed on the basis of NMR and qNMR measurements. For identification, mandatory ^13^C and ^1^H shifts should be compared with databases (e.g., ACDLabs) values available in the literature for the same deuterated solvents.

For some compounds, identification/quantification can be achieved with commercial enzymatic kits, and details concerning the references of the kits should be provided.
